# Inhibition of JMJD6 by 2‐Oxoglutarate Mimics

**DOI:** 10.1002/cmdc.202100398

**Published:** 2021-11-16

**Authors:** Md. Sailful Islam, Cyrille C. Thinnes, James P. Holt‐Martyn, Rasheduzzaman Chowdhury, Michael A. McDonough, Christopher J. Schofield

**Affiliations:** ^1^ Department of Chemistry University of Oxford Chemistry Research Laboratory The Department of Chemistry and the Ineos Oxford Institute for Antimicrobial Research 12 Mansfield Road OX1 3TA Oxford UK

**Keywords:** JMJD6, JmjC hydroxylase / demethylase, prostate cancer, 2-oxogluturate / α-ketoglutarate, oxygenase inhibition, epigenetics, isocitrate dehydrogenase, 2-hydroxygluturate, hypoxia, HIF, PHD inhibitors.

## Abstract

Studies on the inhibition of the human 2‐oxoglutarate dependent oxygenase JMJD6, which is a cancer target, by 2‐oxoglutarate mimics / competitors, including human drugs, drug candidates, and metabolites relevant to cancer are described. JMJD6 assays employed NMR to monitor inhibitor binding and use of mass spectrometry to monitor JMJD6‐catalysed lysine hydroxylation. Notably, some clinically applied prolyl hydroxylase inhibitors also inhibit JMJD6. The results will help enable the development of inhibitors selective for human oxygenases, including JMJD6.

## Graphical abstract

**Selective for human oxygenases**: 2‐Oxoglutarate‐dependent oxygenases such as JMJD6 are targets for treatment of diseases including cancer and anemia. NMR and MS assays on JMJD6, a prostate cancer target, identify 2‐oxoglutarate competitors, including human drugs and drug candidates, as JMJD6 inhibitors. Notably, some clinically applied HIF prolyl hydroxylase inhibitors also inhibit JMJD6. The results will help enable the development of selective inhibitors for human oxygenases, including JMJD6.

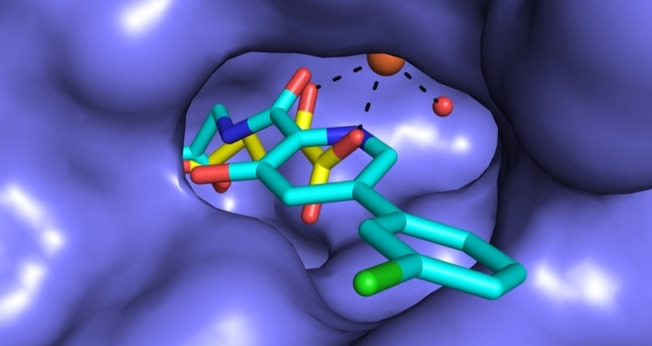

2‐Oxoglutarate (2OG) and Fe(II) dependent oxygenases are established agrochemical targets and more recently have been validated as human drug targets.[^1^, ^2^] The 60–70 human 2OG oxygenases have varied roles, including in collagen biosynthesis, lipid metabolism, ribosome modification, and epigenetic / transcriptional regulation.^[3]^ In humans they catalyse both hydroxylations and histone N^ϵ^‐methyl lysine residue demethylations, the latter catalysed by the JmjC demethylases (KDMs).^[4]^ They play key roles in the hypoxic response in animals by catalysing hydroxylation of the hypoxia inducible factors (HIFs), reactions that modulate HIF activity and signal for its degradation.^[5]^ The activity of the HIF prolyl hydroxylases (PHDs) signals for HIF‐α degradation and is limited by dioxygen availability in cells, a property that enables a graded response to hypoxia. Erythropoietin levels are regulated by HIF and the PHDs are current drug targets for treatment of hypoxia related diseases such as anaemia.[^6^, ^7^, ^8^] PHD inhibitors have been approved for clinical use in China and Japan, but in some cases cardiac effects on heart function have been reported, as was the case in earlier work with collagen prolyl hydroxylase inhibitors.[^7^, ^8^, ^9^] It is presently unclear if this toxicity is due to on or off‐target effects. It is thus important to explore the roles and inhibition profiles of other human 2OG oxygenases.

JMJD6 is a JmjC fold type 2OG oxygenase that is important in human development and which is conserved in ‘early’ eukaryotes.[^10^, ^11^, ^12^, ^13^] JMJD6 is reported to modify proteins including histones and splicing regulatory proteins, in the latter case via lysyl *C*‐5 hydroxylation.[^10^, ^11^, ^12^, ^13^] JMJD6 is also reported to be a histone N‐methyl arginine‐residue demethylase, though this activity needs to be further validated.[^11^, ^12^, ^14^] Although JMJD6 may have roles other than in RNA splicing, evidence has recently been reported that it has a role in regulating production of the V7 splice variant of the androgen receptor (AR).^[15]^ JMJD6 inhibition is thus a potential target for treatment of prostate cancer and has been proposed as a target for ovarian cancer.[^15^, ^16^] Since regulation of the AR is linked to cardiac disease, it is possible JMJD6 inhibition by PHD or other 2OG oxygenase inhibitors could be relevant in vivo.^[17]^

In many cancer cells mutations to metabolic enzyme genes correlates with elevated levels of acidic metabolites, including succinate, fumarate, and 2‐hydroxyglutarate (2HG), which in turn are proposed to lead to inhibition of 2OG oxygenases (and other enzymes), in a manner promoting tumorigenesis / cancer development.^[18]^ JMJD6 has not been investigated in this regard. Indeed, few studies have been reported on JMJD6 inhibition, in particular using its validated lysyl‐hydroxylase activity (Figure 1).[^11^, ^12^, ^13^, ^16^, ^19^, ^20^]

**Figure 1:**
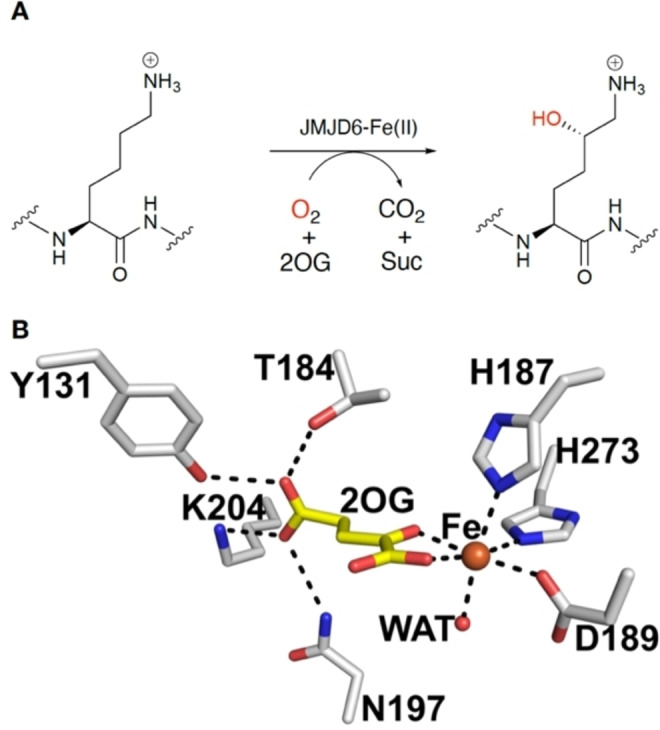
JMJD6 lysyl‐hydroxylase activity is dependent on Fe(II) and 2‐oxoglutarate (2OG). (A) JMJD6 catalyzes lysine residue hydroxylation to produce L‐hydroxy‐(5*S*)‐lysine. (B) View from a JMJD6 crystal structure (PDB: 6GDY) showing the active site (white sticks) with Fe (orange sphere) and 2OG (yellow) bound.

Here we report the results of NMR and MS based assays with JMJD6 with a range of potential inhibitors, including 2OG mimics, clinically applied 2OG oxygenase inhibitors, and naturally occurring metabolites. The results should help to enable the development of improved inhibitors of both JMJD6 and other human 2OG oxygenases.

To monitor inhibition of JMJD6 by a set of potential 2OG competitors / known 2OG oxygenase inhibitors, we employed an assay using matrix assisted laser desorption ionisation time of flight (MALDI‐TOF) mass spectrometry (MS), with a twelve‐residue fragment (NPKRSRSREHRR, with a *C*‐terminal amide) of the splicing regulatory protein LUC7L2_267‐278_, which we have used in previous studies on JMJD6.^[12]^ In selected cases we validated competition between the inhibitors and 2OG for binding to JMJD6 using a ligand‐observed NMR based assay as previously used for studies with PHD2 and γ‐butyrobetaine hydroxylase i. e., the inhibitors were tested for their ability to displace 2OG from the JMJD6^Δ363‐403^.Zn(II)).2OG complex.[^12^, ^21^, ^22^]

Initially, we used the MS assay to screen a range of potential JMJD6 inhibitors, based on scaffolds identified as inhibitors of other 2OG oxygenases, grouped into: (**i**) TCA cycle and related acidic compounds; (**ii**) 2OG mimics / broad spectrum 2OG oxygenase inhibitors; (**iii**) PHD inhibitors in clinical use / investigation; (**iv**) tricarbonyl type compounds, which are known iron oxygenase inhibitors; (**v**) reported inhibitors of the JmjC KDMs.^[23]^ Five of these compounds inhibited JMJD6^Δ363‐403^ activity by 95 % or more, four by 90–95 %, and an additional eight by 60–90 % (Figure S1).^[1]^ None of the tested JmjC KDM inhibitors were potent JMJD6 inhibitors. Thirty‐six compounds were selected for IC_50_ determination (Table 1, Figures 2, S2–S5). The 8‐hydroxyquinoline derivatives 5‐carboxy‐8‐hydroxyquinoline (IOX1) and **2**, pyridine derivatives pyridine‐2,4‐dicarboxylate (2,4‐PDCA) and 2,2’‐bipyridine‐4,4’‐dicarboxylate (2,4‐BPDCA), and the PHD inhibitors GSK1278863, Vadadustat and AKB‐6899 were the most potent identified inhibitors (IC_50_ values <15 μM) (Table 1). By contrast with 2,4‐PDCA, another widely used 2OG analogue, N‐oxalylglycine (NOG), gave a higher IC_50_ value (296 μM).^[1]^

**Table 1:** IC_50_ values for JMJD6 inhibitors. IC50 values (mean±standard deviation, n=3) were determined by MALDI‐TOF MS assays using 10 μM JMJD6Δ363‐403, 100 μM LUC7L2267‐278 (NPKRSRSREHRR), 100 μM (NH4)2Fe(SO4)2.6H2O, 400 μM L‐sodium ascorbate, 20 μM 2OG,^[12]^ with varied inhibitor concentrations (0–10 mM). K_D_^app^ values are given in parentheses for selected compounds.

	IC_50_ [μM] (K_D_^app^ [μM])		IC_50_ [μM] (K_D_^app^ [μM])
*TCA Cycle Intermediates*		*PHD Inhibitors*	
Citrate	987±1.3	FG‐4592	23±1.5
d‐2HG	622±1.4	GSK1278863	10±1.2
Fumarate	165±1.4 (109±22.0)	Molidustat	74±1.6
Isocitrate	1055±1.5	Vadadustat	7±1.6 (14±5.5)
L‐2HG	871±1.5	AKB‐6899	9±1.5 (3±2.8)
Malate	5103±2	*Tricarbonyl Compounds*	IC_50_ (μM)
Pyruvate	2377±1.7		
Oxaloacetate	204±2.0	**4**	175±1.5
Succinate	261±2.3 (159±28.8)	**5**	19±1.3
*2OG Analogues*	IC_50_ [μM] (K_D_^app^ [μM])	**6**	23±1.2
		**7**	43±1.1
NOG	296±1.9	**8**	27±1.4
**1**	189±1.5	**9**	≥1000
2,4 PDCA	13±1.3 (6±1.3)	**10**	≥1000
2,4 BPDCA	6±1.6 (7±1.8)	**11**	67±1.2
IOX1	10±1.5	**12**	58±1.4
**2**	5±1.3	**13**	135±1.3
Daminozide	94±1.5	**14**	163±1.3
**3**	25±2.0	**15**	≥1000
		**16**	214±1.4
		**17**	22±1.4

**Figure 2:**
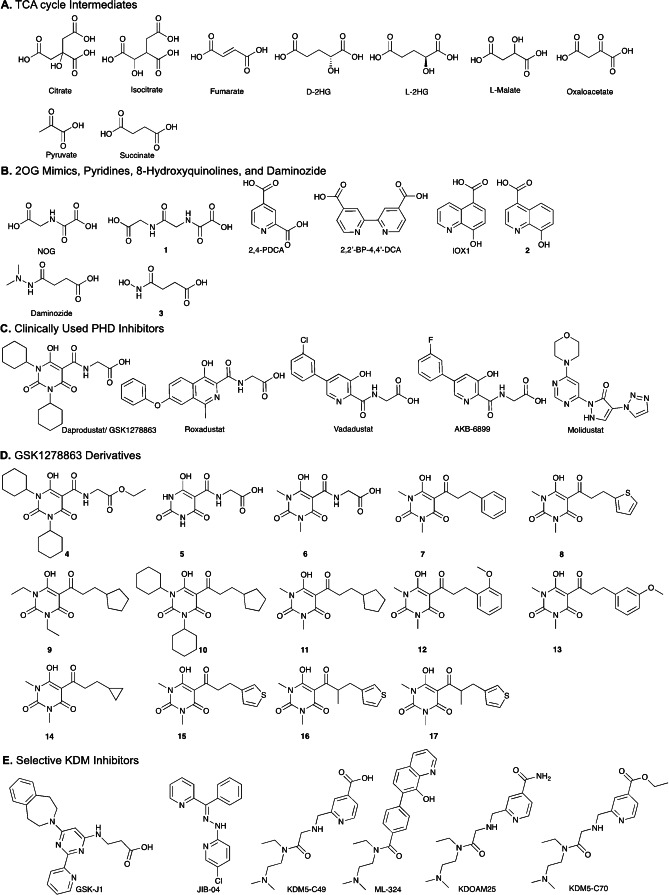
Potential Inhibitors of JMJD6 studied in our work.

We investigated whether the inhibitors compete for binding to JMJD6^Δ363‐403^, using ^1^H NMR to investigate the extent to which they displace 2OG from the JMJD6^Δ363‐403^.Zn(II).2OG complex, initially with a fixed inhibitor concentration (Figure S6).[^21^, ^22^] The results imply the analysed inhibitors compete with 2OG and broadly, though not fully, correlate with the IC_50_ values; in the initial screen succinate, fumarate, 2,4‐PDCA, 2,4‐BPDCA, Vadadustat and AKB‐6899 most efficiently displaced 2OG of the tested compounds. The correlation with IC_50_ values was less clear in the case of the tricarbonyl type inhibitors.

Apparent binding constant K_D_^app^ values for six selected inhibitors were determined by NMR with values of 159 μM / succinate, 109 μM / fumarate, 6 μM / 2,4‐PDCA, 7 μM / 2,4‐BPDCA, 3 μM / Vadadustat, and 14 μM / AKB‐6899, being obtained (Figures S7–S9, Table 1). Although, in some cases the errors were relatively high, the calculated relative K_D_^app^ values of these are broadly consistent with the IC_50_ values (Table 1), e. g., the close 2OG analogue NOG which was a poor inhibitor only weakly displaced 2OG (Figure S6). Daminozide, a selective inhibitor of certain JmjC KDMs, showed weak inhibition of JMJD6 (IC_50_ 94 μM), but the structurally related succinyl hydroxamic acid derivative **3**, which is a broad‐spectrum inhibitor of JmjC KMDs, was a more potent JMJD6 inhibitor (IC_5*0*_ 25 μM, Table 1).^[24]^

Both the biological roles and biochemistry of JMJD6 presently appear rather complex; thus, the results presented here should be regarded as preliminary in relation to their relevance to research and medicinal applications of 2OG oxygenase inhibitors. It should also be noted that there is likely scope for optimisation of our current JMJD6 assay (which employs a JMJD6^Δ363‐403^: LUC7L2_267–278_ ratio of 1 : 10), however, the IC_50_ values give an indication of relative potency.^[12]^ The results identify the pyridine‐carboxylate and 8‐hydroxyquinoline scaffolds as suitable ring systems for optimisation into potent JMJD6 inhibitors, as has been done for some other 2OG oxygenases; hydroxamates also show potential as JMD6 inhibitors.^[25]^ Interestingly, the close 2OG analogue NOG was not a potent JMJD6 inhibitor in our assays.^[1]^

The results suggest that cellular studies employing pyridine‐carboxylate and 8‐hydroxyquinoline ring type 2OG oxygenase inhibitors should take into account the possibility of JMD6 inhibition. Indeed, 2,4‐PDCA has been used to inhibit prostate cancer splicing in cells (AR V7 variant of the androgen receptor).^[15]^ The IC_50_ values for the PHD inhibitors causing are particularly notable given that these compounds are in clinical use or have been in clinical development. Vadadustat, AKB‐6899, and GSK1278863 were amongst the most potent JMJD6 inhibitors identified by us (Table 1). Thus, the possibility of PHD inhibitors of JMJD6 inhibition during their clinical application should be considered. However, given the complexity of JMJD6 biology this is by no means certain, and we appreciate the results with isolated JMJD6 presented here are not necessarily representative of in vivo potencies. Indeed, it is quite likely the potencies of JMJD6 inhibition will vary with respect to different JMJD6 substrates and possibly different (post‐translationally modified) forms of JMJD6 itself.[^11^, ^13^]

The potency of JMJD6 inhibition by TCA and related intermediates, whose concentrations are sometimes elevated in cancer, was moderate at most, with fumarate being the most potent of these compounds identified as inhibiting JMJD6. The high‐levels of ‘oncometabolites’, e. g., 2‐hydroxysuccinate and fumarate, reached in some cancer cells suggest it is possible that JMJD6 inhibition is relevant to their roles in tumorigenesis, but the current biochemical evidence suggest that other 2OG oxygenases may be more potently inhibited by them than JMJD6 in cells, though lack of detailed knowledge on subcellular concentrations of enzymes and oncometabolites makes answering this question difficult.[^18^, ^23^, ^26^]

In conclusion, together with recently reported studies employing succinate production and computational methods coupled with cellular studies,[^16^, ^19^, ^20^] the lysyl‐hydroxylation based assays and results presented here imply JMJD6 is amenable to inhibition by small‐molecules, in particular 2OG competitors, including some clinically used compounds.[^16^, ^19^] Future work can be directed towards optimisation of the 2OG competing inhibitors described here to make potent and selective JMJD6 inhibitors for use in functional assignment and target validation work on JMJD6 for cancer treatment. The JMJD6 assays described may also help in the development of selective inhibitors of other human 2OG oxygenases, including of the PHDs.

## Conflict of interest

The authors declare no conflict of interest.

## Supporting information

As a service to our authors and readers, this journal provides supporting information supplied by the authors. Such materials are peer reviewed and may be re‐organized for online delivery, but are not copy‐edited or typeset. Technical support issues arising from supporting information (other than missing files) should be addressed to the authors.

Supporting Information
