# Supplementary material for: Inhibition of JMJD6 by 2‐Oxoglutarate Mimics
Source: ChemMedChem. 2021 Nov 16;17(1):e202100398. doi: 10.1002/cmdc.202100398 (PMC9299220; doi:10.1002/cmdc.202100398)
Supplement: Supplementary file 1 — Supporting Information [file CMDC-17-0-s001.pdf]

ChemMedChem

Supporting Information

Inhibition of JMJD6 by 2-Oxoglutarate Mimics

Md. Sailful Islam, Cyrille C. Thinnes, James P. Holt-Martyn, Rasheduzzaman Chowdhury, Michael A. McDonough, and Christopher J. Schofield*

Supporting Information:

Figure S1	Initial inhibition assays
Figure S2	IC_{50} curves for TCA cycle intermediates / related compounds
Figure S3	IC_{50} curves for 2OG analogues
Figure S4	IC_{50} curves for PHD inhibitors
Figure S5	IC_{50} curves for tricarbonyl type compounds
Figure S6	2OG displacement ligand-binding assay with JMJD6 via CPMG-edited ^{1}H NMR analysis
Figure S7	K_D^{app} determination for TCA cycle intermediates binding to JMJD6
Figure S8	K_D^{app} determination for pyridine derivatives binding to JMJD6.
Figure S9	K_D^{app} determination for selective PHD inhibitors binding to JMJD6
	Conditions for NMR assays

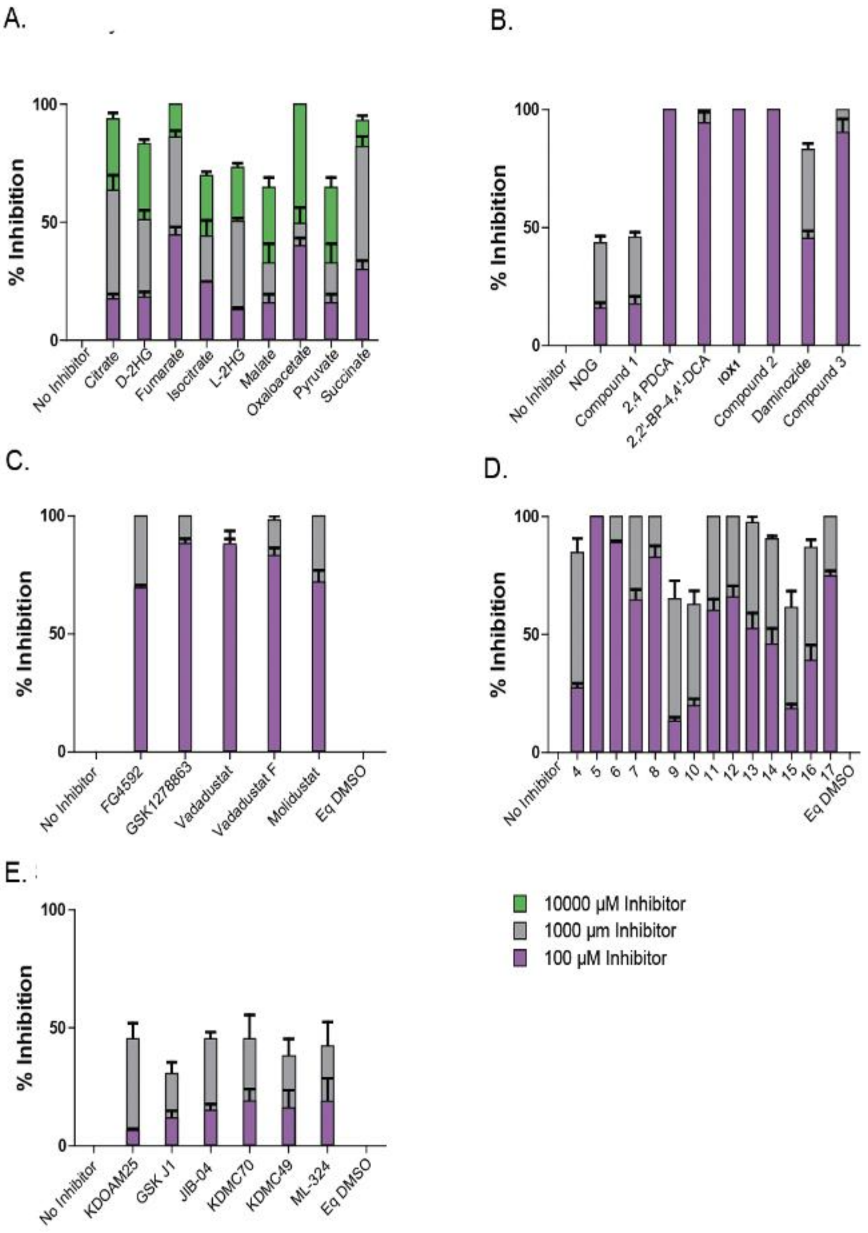

Figure S1. Initial inhibition assays. Bar charts showing extent of JMJD6 inhibition by 0.1, 1, and 10 mM compound: (**A**) TCA cycle intermediates/related compounds, (**B**) 2OG analogues, (**C**) PHD inhibitors, (**D**) Tricarbonyl type compounds, (**E**) JmjC KDM inhibitors. Values represent the mean ± SD (n = 3). Conditions: 10 µM JMJD6$^{\Delta 363\text{-}403}$ (prepared as reported [11]) and 100 µM LUC7L2$_{267\text{-}278}$ (NPKRSRSREHRR, with a C-terminal amide) at 37 °C in 50 mM HEPES pH 7.5 buffer with 100 µM $(NH_4)_2Fe(SO_4)_2.6H_2O$ (Sigma-Aldrich), 400 µM L-sodium ascorbate (Sigma-Aldrich), and 24 µM 2OG (K_m of 2OG) (Fluka) with varied inhibitor concentrations (0-10 mM). Reactions were quenched and products analysed by MALDI-TOF MS following a reported procedure.[11]

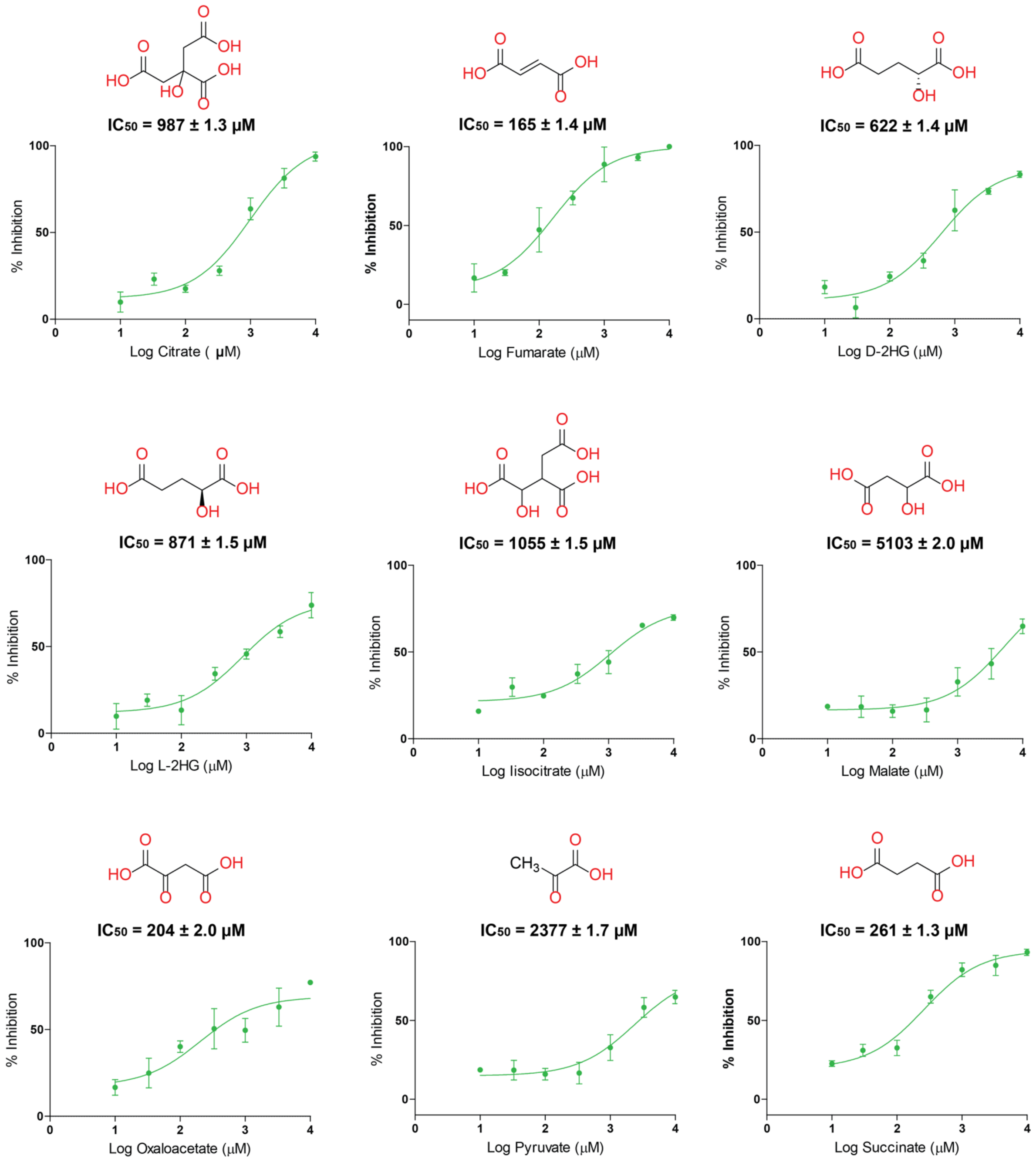

Figure S2. **IC_{50} curves for TCA cycle intermediates /related compounds**. Conditions: see Figure S1 legend. Values represent the mean ± SD (n = 3).

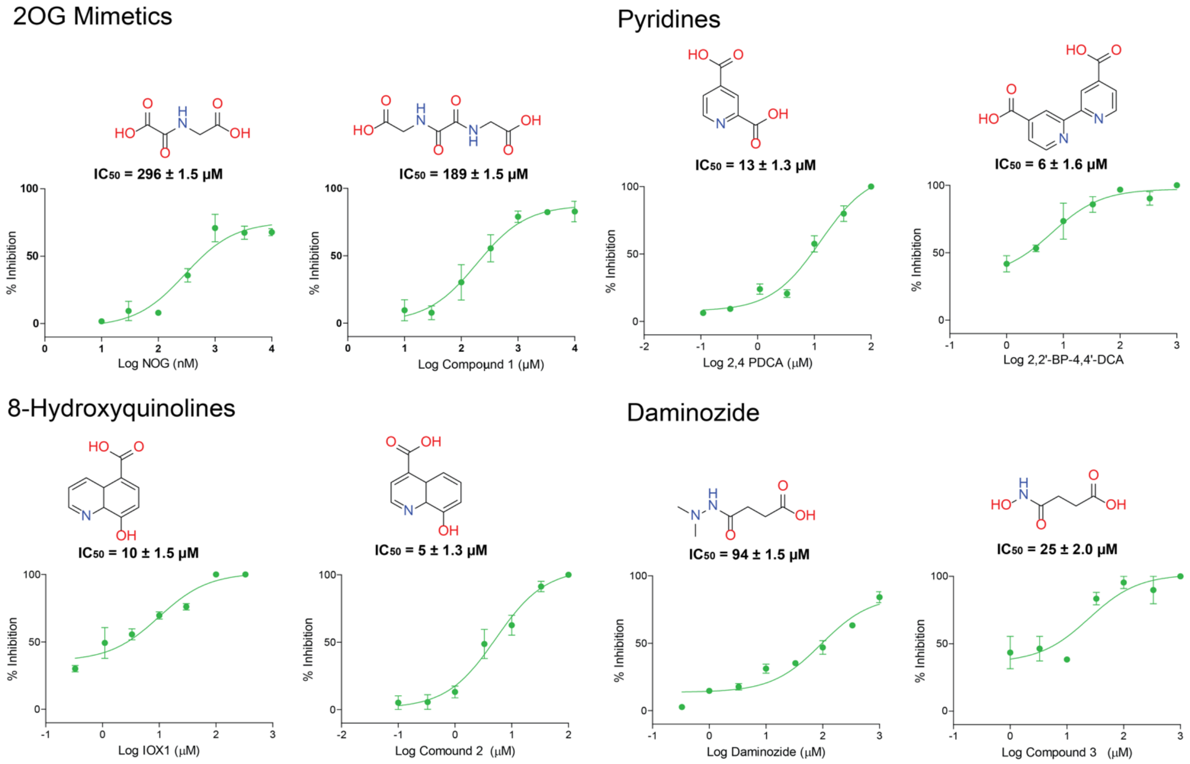

Figure S3. **IC_{50} curves for 2OG analogues**. Conditions: see Figure S1 legend. Values represent the mean ± SD (n = 3).

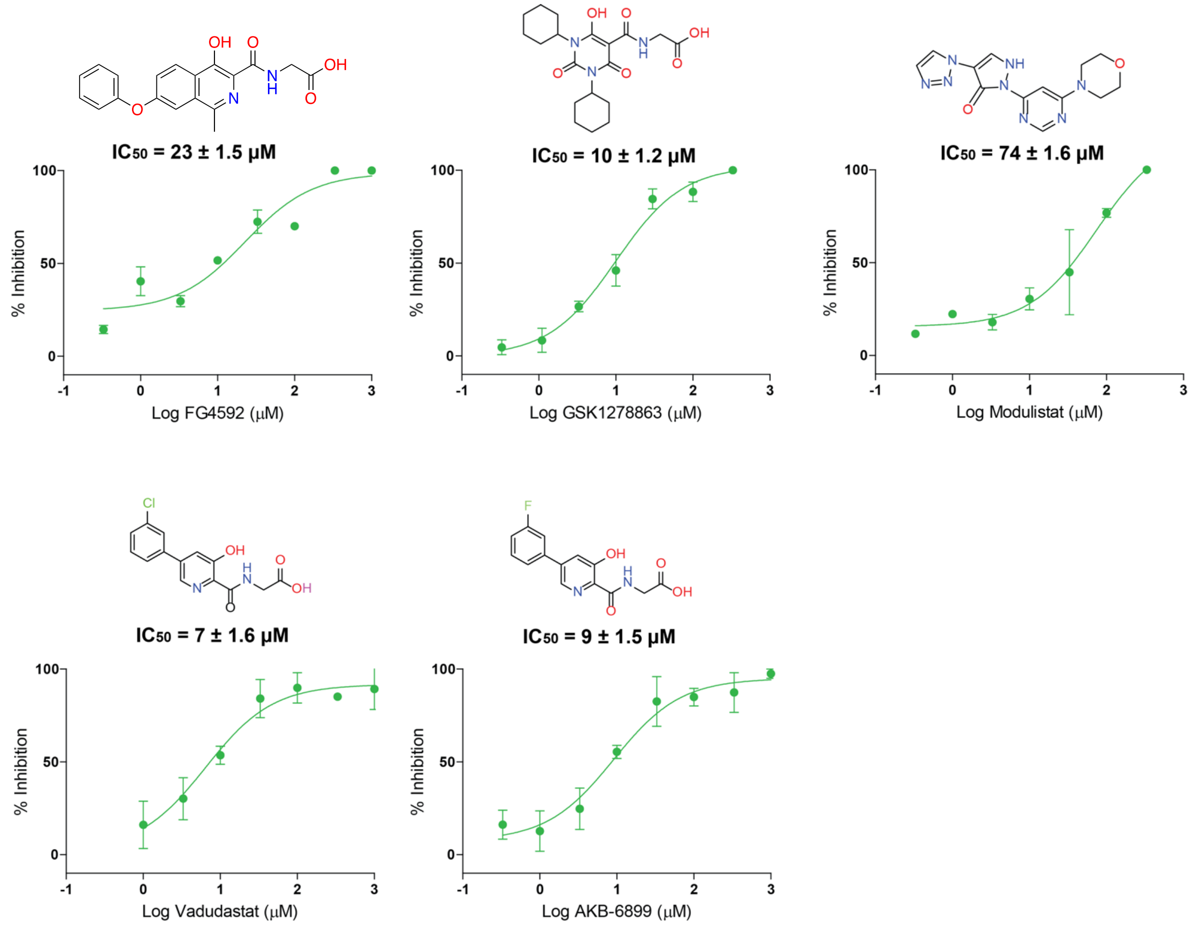

Figure S4. IC_{50} curves for PHD inhibitors. Conditions: see Figure S1 legend. Values represent the mean ± SD (n = 3).

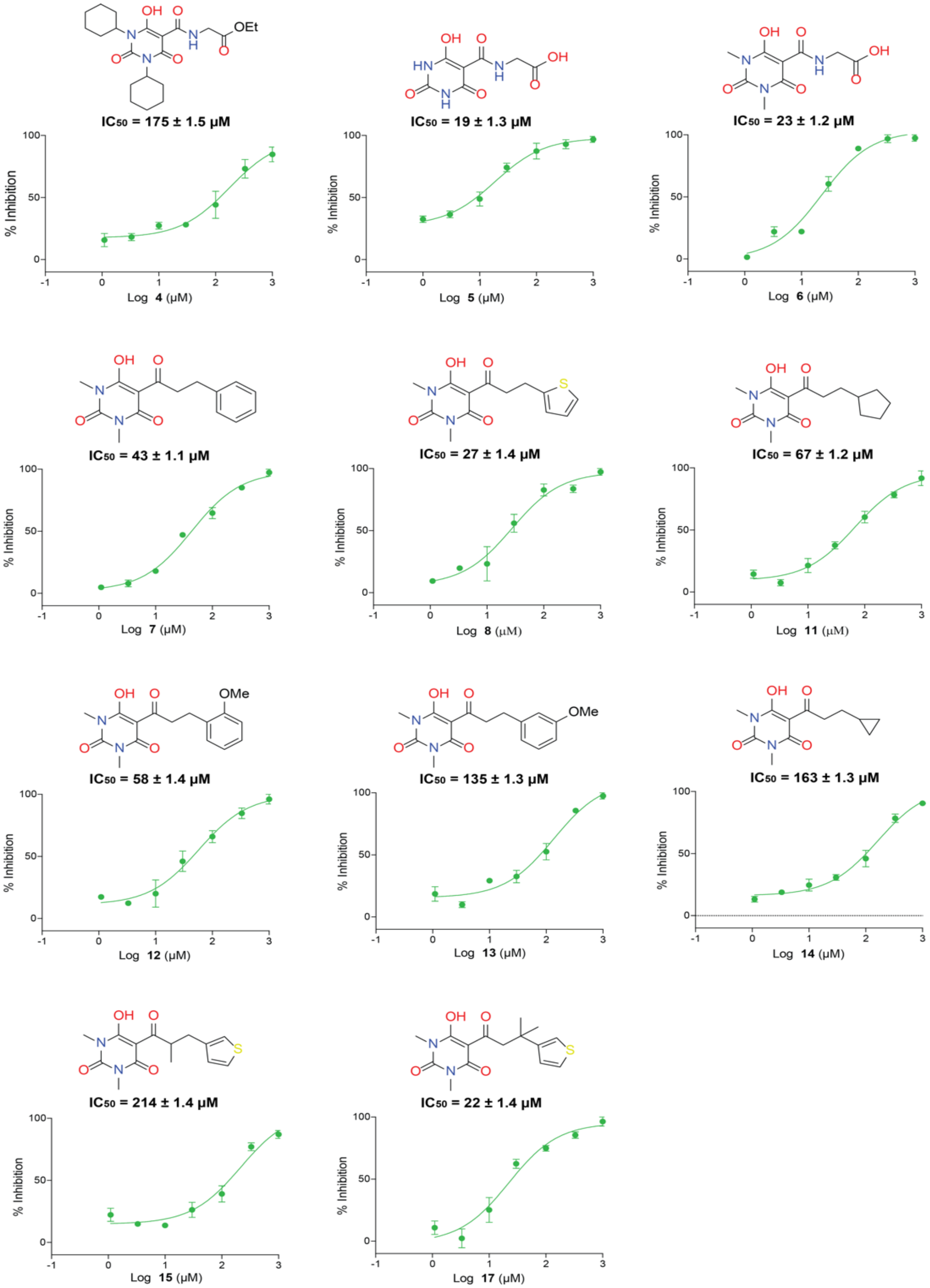

Figure S5. ***IC50*** **curves for tricarbonyl compounds**. Conditions: see Figure S1 legend. Values represent mean ± SD (n = 3).

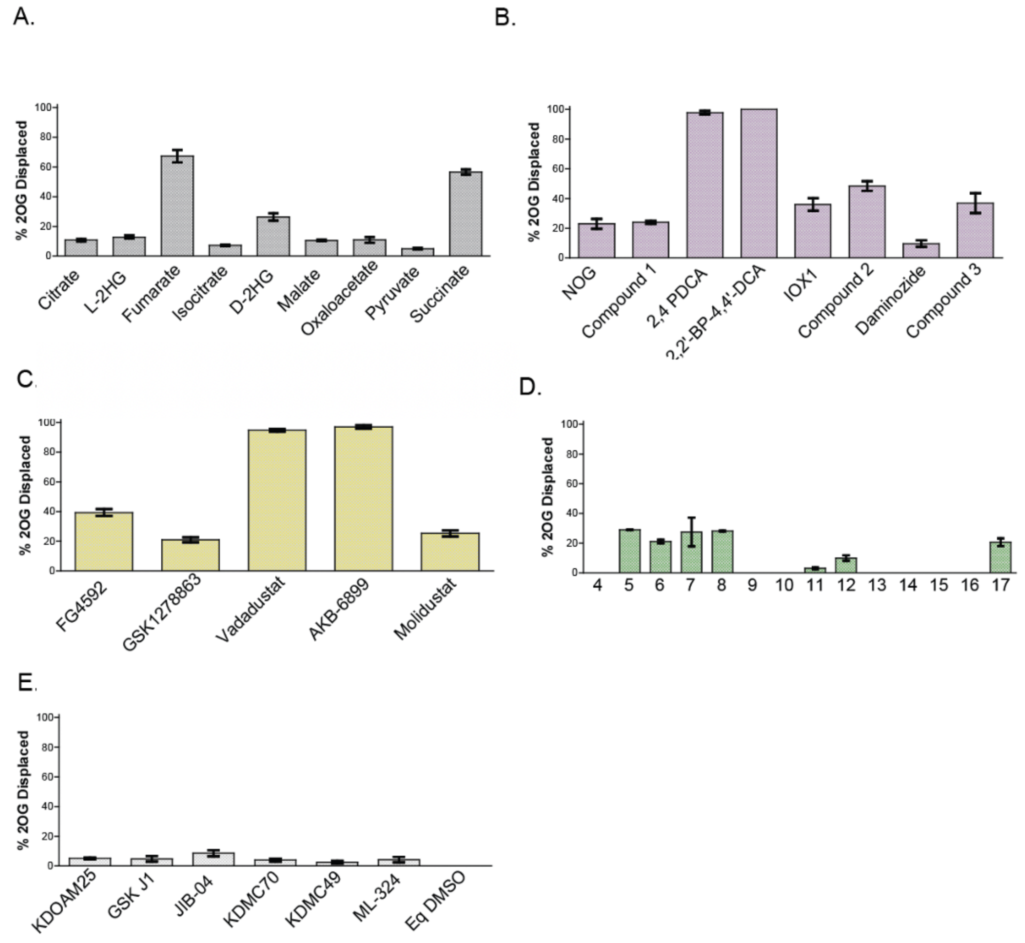

Figure S6: 2OG displacement ligand-binding assays with JMJD6 using CPMG-edited ^{1}H NMR. Bar charts show the extent of 2OG displacement for: (**A**) TCA cycle intermediates, (**B**) 2OG mimetics, (**C**) PHD inhibitors, (**D**) tricarbonyl type compounds, (**E**) KDM inhibitors. Conditions: 60 µM EDTA-treated JMJD6$^{\Delta 363\text{-}403}$, 200 µM Zn(II), 10 µM 2OG, and 400 µM of the ligand, all in 50 mM Tris-D11, pH 7.0 in 10% D_2O. Values represent the mean (n = 3) ± standard deviation (SD). Eq DMSO: DMSO control without inhibitor. Structures for compounds in A-D are given in Figure 2.

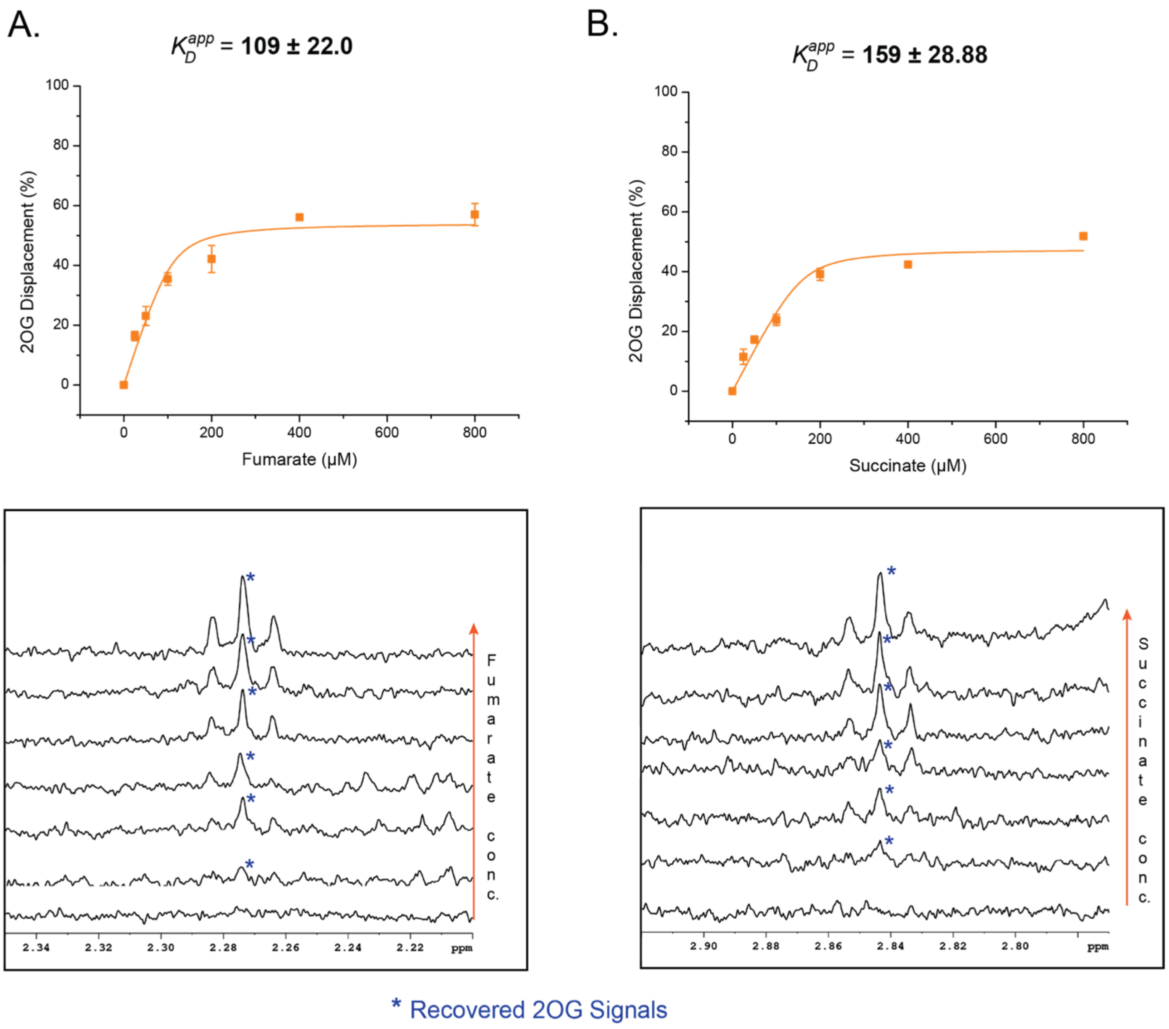

Figure S7: ***K_D*^{app} determination for TCA cycle intermediates / related compounds binding to JMJD6.** Plots used for the determination of K_D^{app} values of (**A**) fumarate and (**B**) succinate. The insets show competition between 2OG and the ligands. Conditions: 60 µM EDTA-treated JMJD6, 200 µM Zn(II), 20 µM 2OG, 0-400 µM ligand, all in 50 mM Tris-D11 pH 7.0 in 10% D_2O. Values represent the mean ± SD (n = 3). Compound structures are given in Figure 2.

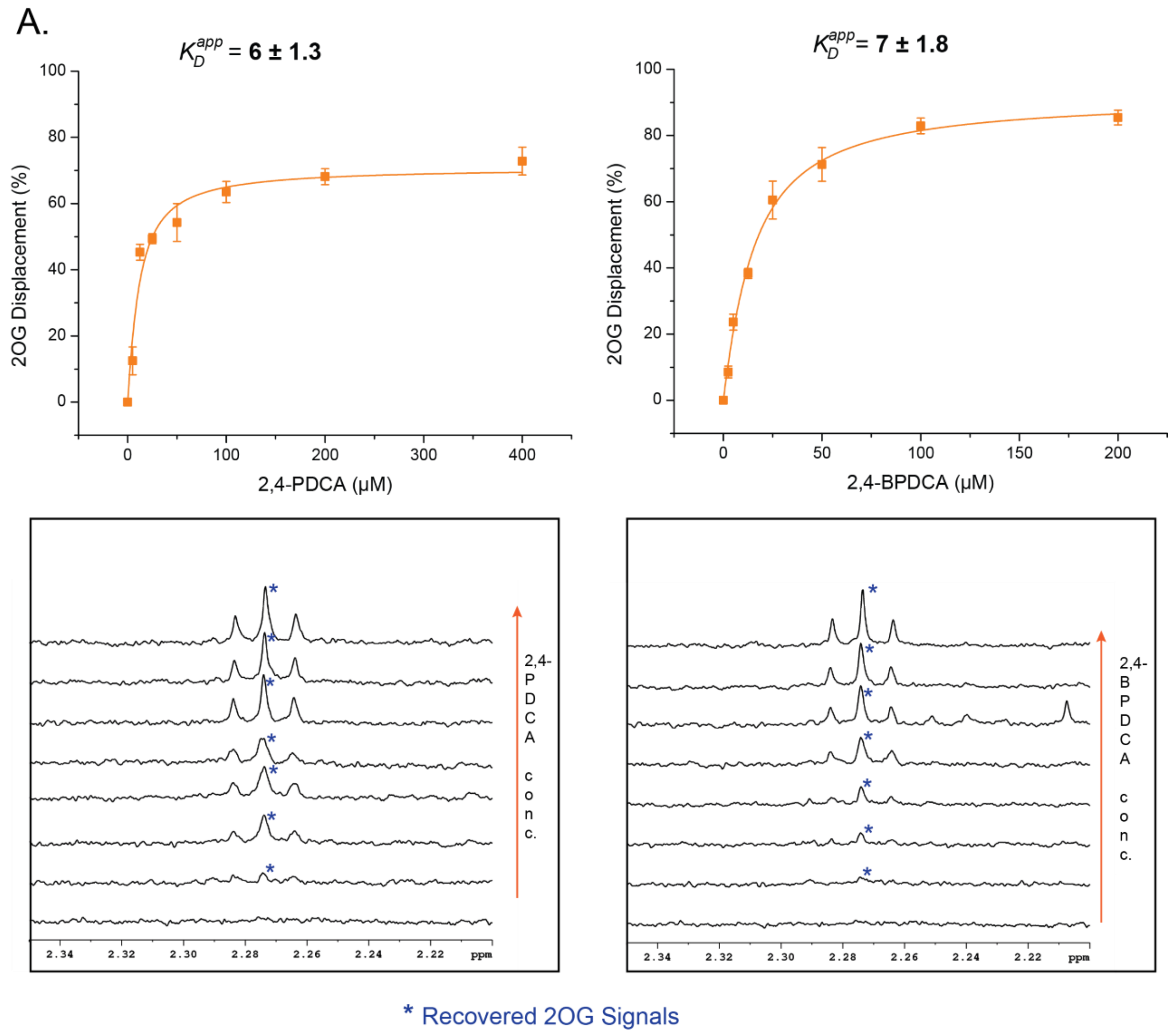

Figure S8: ***K_D*^{app} determination for pyridine derivative binding to JMJD6.** Plots used for determination of K_D^{app} values (**A**) 2,4-PDCA and (**B**) 2,4-BPDCA. The spectra show evidence for competition between 2OG and the ligands. Conditions: 60 µM EDTA-treated JMJD6, 200 µM Zn(II), 20 µM 2OG, 0-800 µM ligand, in 50 mM Tris-D11 pH 7.0 in 10% D_2O. Values represent the mean ± SD (n = 3). Compound structures are given in Figure 2.

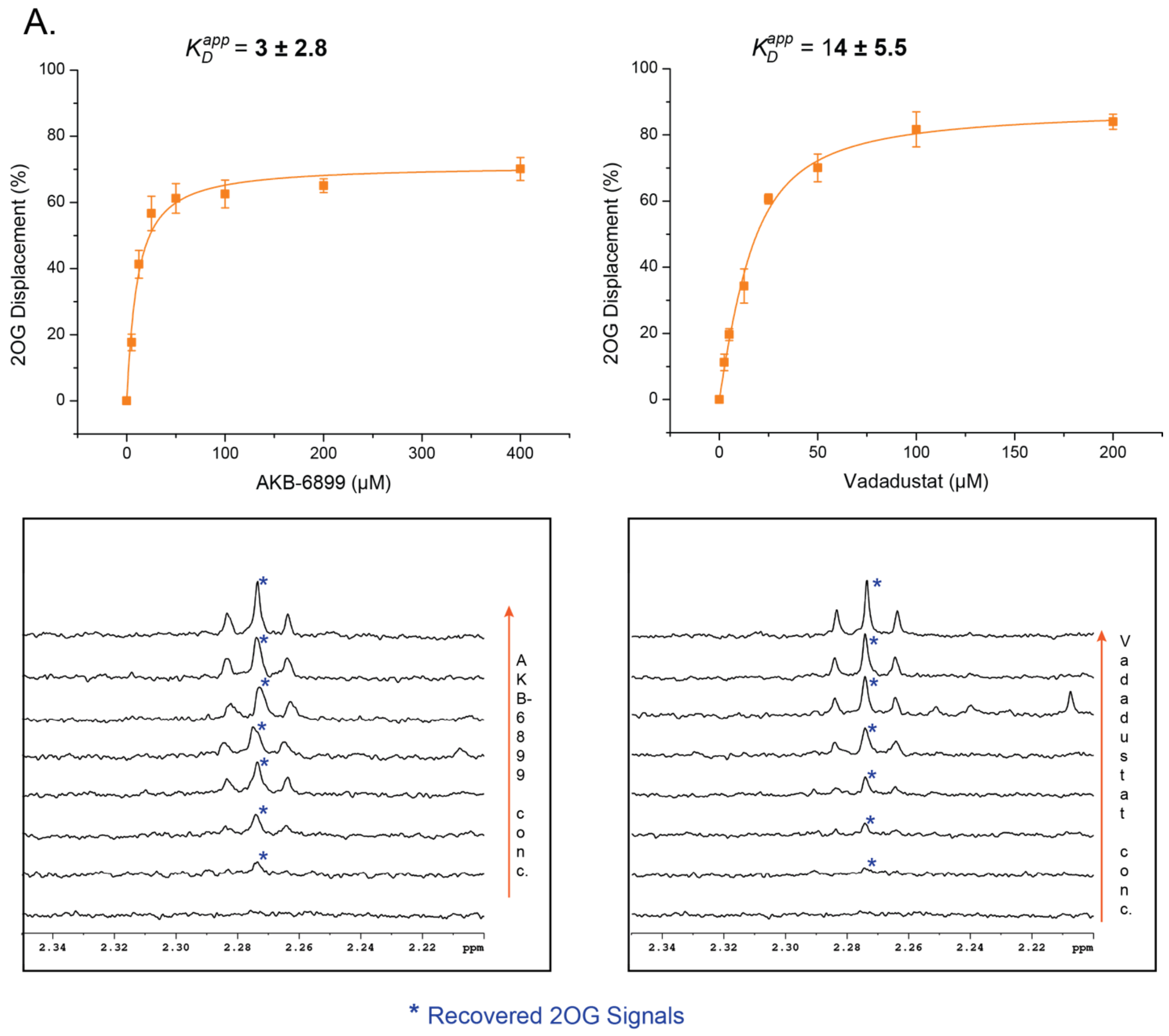

Figure S9: ***K_D*^app^ determination for PHD inhibitor binding to JMJD6.** Plots used for the determination of K_D^{app} value (**A**) AKB-6899 and (**B**) Vadadustat. The spectra show values for competition between 2OG and the ligands. Conditions: 60 µM EDTA-treated JMJD6, 200 µM Zn(II), 20 µM 2OG, 0-400 µM ligand, in 50 mM Tris-D11 pH 7.0 in 10% D_2O. Values represent the mean ± SD (n = 3). Compound structures are given in Figure 2.

Conditions for NMR assays

NMR was used to measure 2OG displacement from JMJD6 caused by inhibitors according to the method of Leung et al. [15] The Carr-Purcell-Meiboom-Gill (CPMG) pulse sequence coupled with periodic refocusing of J evolution by coherence transfer (PROJECT) method was applied for spectral editing was used to edit out protein signals. In the initial screen (Figure S1), 40 µM JMJD6$^{\Delta 363\text{-}403}$ [JMJD6$^{\Delta 363\text{-}403}$.Zn(II)] was added to a mixture of 20 µM 2OG and 400 µM inhibitor; the ^{1}H NMR-spectra of 2OG were then analysed. To determine the apparent binding constant (K_D^{app}), 20 µM of 2OG was titrated against increasing concentrations of JMJD6$^{\boldsymbol{\Delta 363\text{-}403}}$ in the presence of Zn(II) [JMJD6$^{\Delta 363\text{-}403}$.Zn(II)]. Thus, JMJD6$^{\Delta 363\text{-}403}$.Zn(II) was added until the methylene signals of 2OG were no longer observed, indicating complete 2OG binding to JMJD6. The extent of 2OG (%) bound to JMJD6$^{\boldsymbol{\Delta 363\text{-}403}}$ was calculated and the values were plotted against the concentration of JMJD6$^{\boldsymbol{\Delta 363\text{-}403}}$ using Origin™. The K_D^{app} of 2OG was calculated as reported.[11] To determine the K_D^{app} for the inhibitor, the recovery of 2OG-signals was monitored in the presence of varied concentrations of inhibitor (0-800 µM).

$$\%\ \text{2OG Displacement} = \frac{I_{2OG} - I_{2OG(0)}}{I_{2OG(blank)} - I_{2OG(0)}} \times 100$$

$$\Delta_{obs} = \Delta_{max} \times \frac{(K_D^{app} + [L]_0 + [P]_0) - \sqrt{\{(K_D^{app} + [L]_0 + [P]_0)^2 - 4 \times [L]_0 \times [P]_0\}}}{2 \times [P]_0}$$

where, I_{2OG} is the integral of the 2OG ^{1}H signal in the presence of both inhibitor and JMJD6$^{\Delta 363\text{-}403}$.Zn(II)], $I_{2OG(0)}$ is the integral of 2OG in the presence of JMJD6$^{\Delta 363\text{-}403}$.Zn(II)] without inhibitor, $I_{2OG(blank)}$ is the integral of 2OG in the absence of both JMJD6$^{\Delta 363\text{-}403}$.Zn(II)] and inhibitor, Δ_{obs} is the change in the integral of 2OG resulting from titration, Δ_{max} represents the maximum change of the monitored NMR parameter (2OG integral), $[L]_0$ is the titrated ligand concentration, and $[P]_0$ is the JMJD6$^{\Delta 363\text{-}403}$concentration. 5-10% error was typically allowed in the curve fitting process.
